# Long noncoding RNA VPS9D1-AS1 promotes esophageal squamous cell carcinoma progression via the Wnt/β-catenin signaling pathway

**DOI:** 10.7150/jca.54556

**Published:** 2021-10-02

**Authors:** Liang Ma, Wenyue Yan, Xingwei Sun, Ping Chen

**Affiliations:** 1 Department of Oncology, Yancheng First Hospital, Affiliated Hospital of Nanjing University Medical School, The First People's Hospital of Yancheng, Yancheng, Jiangsu , China.; 2 Department of Intervention, The Second Affiliated Hospital of Soochow University, Suzhou, Jiangsu, China.

**Keywords:** VPS9D1-AS1, long non-coding RNA, prognosis, Wnt/β-catenin, esophageal squamous cell carcinoma

## Abstract

The VPS9D1 antisense RNA1 (VPS9D1-AS1, lncRNA MYU) can act as an oncogene or an antioncogene in different malignancies. In the present study, we demonstrated that VPS9D1-AS1 is significantly upregulated in esophageal squamous cell carcinoma (ESCC) and assessed its biological function and clinical prognosis. RNA-sequencing was conducted in four pairs of ESCC tissues and normal adjacent tissues (NATs). Compared with controls, lncRNA VPS9D1-AS1 was highly expressed in ESCC tissues, cell lines and plasma. VPS9D1-AS1 upregulation significantly correlated with the histopathological grade and clinical stage of ESCC. Analyses revealed poor prognosis in ESCC patients with high VPS9D1-AS1 expression. VPS9D1-AS1 knockdown led to the inhibition of tumor proliferation, migration, and invasion *in vivo* and vitro. VPS9D1-AS1 silencing downregulated the Wnt/β-catenin signaling pathways by acting on key proteins such as β-catenin and c-Myc. However, the expressions of these proteins increased after the addition of pathway agonist CT99021. Therefore, taken together VPS9D1-AS1 is highly expressed in ESCC and its expression can lead to poor prognosis. In conclusion, this study suggested that VPS9D1-AS1 acts as a vital part in facilitating ESCC progression and can be a potential biomarker for the diagnosis of patients with ESCC.

## Introduction

Esophageal cancer has become the primary cause of cancer related deaths in China owning to its high incidence and low survival rate. Esophageal squamous cell carcinoma (ESCC) and adenocarcinoma are the two primary types of esophageal cancer. In China, ESCC is more frequent than adenocarcinoma. Although advancements in medical technology have resulted in some progress in the diagnosis and treatment of ESCC, the 5-year survival rate has not been significantly improved 1-2. Owning to increasing incidence and worse prognosis, researchers are beginning to focus on the early diagnosis and treatment of ESCC.

More and more noncoding RNAs (ncRNAs) are generally considered as regulators of tumorigenesis. Long noncoding RNAs (lncRNAs) are a kind of ncRNAs that are longer than 200 nucleotides and that lack or have no open reading coding frame. The number of lncRNAs is much lower than that of mRNAs; they are generally expressed in low levels in tissues and body fluids and have higher tissue and organ specificity 3. Previous studies have reported a series of aberrant lncRNA expressions in patients with ESCC; these events were related to certain biological characteristics such as proliferation and invasion of ESCC 4-7. Therefore, it is important to identify tumor related lncRNAs and elucidate the regulatory networks. This will result in a deeper understanding of the tumorigenic mechanisms as well as provide effective cancer diagnosis and development of cancer therapeutics.

## Materials & methods

### RNA-sequencing

Four pairs of ESCC tissues and normal adjacent tissues (NATs) were obtained from patients during surgery at Yancheng First Hospital, Affiliated Hospital of Nanjing University Medical School, Yancheng, Jiangsu, China. All samples were immediately stored in liquid nitrogen and transported to the designated biotechnology company (Gminix, Shanghai, China). The RNA-sequencing was performed using the Illumina platform by Genminix Informatics co. Ltd in Shanghai.

### Tissue and plasma sample collection

A total of 92 patients with ESCC who were admitted to Yancheng First Hospital, Affiliated Hospital of Nanjing University Medical School between January 2015 and June 2016 were included in this study. In particular, NATs were collected at least 5 cm away from the tumor tissues. Inclusion criteria of patients with ESCC: 1. Patients aged 18-70 years, diagnosed with primary ESCC by digestive endoscopy and pathology; 2. There are indications for radical resection of ESCC or palliative surgery but no obvious contraindications to surgery. Exclusion criteria: 1. Patients with major basic diseases such as heart and lung system that seriously affect daily life and actions; 2.Have autoimmune deficiency diseases such as AIDS or are receiving long-term systemic steroid treatment; 3.Complicated with other malignant tumors; 4. Patients with active chronic hepatitis, active tuberculosis and other infectious diseases; 5.Any known mental illness or substance abuse disorder; 6.Received any form of radiation, chemotherapy, or tumor targeting and immunotherapy. Plasma samples were collected from 25 patients with ESCC before surgery or chemoradiotherapy and from 35 patients with benign esophageal lesions between January 2018 and June 2019. Inclusion and exclusion criteria of these 25 patients with ESCC were the same as before. Inclusion criteria of patients with benign esophageal lesions: 18-70 years old, benign esophageal diseases including chronic esophagitis, esophageal leiomyoma, achalasia of cardia, et al. Exclusion criteria was the same as patients with ESCC. The Research Ethics Committee of Yancheng First Hospital, Affiliated Hospital of Nanjing University Medical School approved the present study. All samples were histologically confirmed.

### Cell culture

Eca109, Kyse150, TE-1, TE-13, and het-1A were purchased from the Chinese Academy of Sciences (Shanghai, China). The cells were kept in RPMI-1640 medium (HyClone, USA) supplemented with 10% fetal bovine serum (Gibco, CA) and cultured with 5% CO_2_ in a 37 °C humidified incubator.

### Quantitative real-time polymerase chain recation (qRT-PCR) assay

Total RNA was extracted from frozen tissues, plasma, and cell cultures using the TRIzol reagent (Thermo Fisher Scientific, USA) and then reverse-transcribed to cDNA using the Revert Aid First Strand cDNA Synthesis Kit 1622 (Thermo Fisher Scientific, USA). qRT-PCR was performed using the ABI® step one plus Real-time PCR system (Applied Biosystems Life Technologies, USA) with the SYBR Green Master Mix (Thermo Fisher Scientific, USA). The primers used are as follows: VPS9D1-AS1: AGCTTTCCTCCTTCATCGGA (forward) and TGGCTTGCAGGGAAAACAC (reverse); GAPDH: GAACGGGAAGCTCACTGG (forward) and GCCTGCTTCACCACCTTCT (reverse) (RiboBio Co., Guangzhou, China).

The relative expression of VPS9D1-AS1 was standardized with that of GAPDH using the 2^-ΔΔCt^ method.

### Plasmid construction and cell transfection

The small hairpin RNA (shRNA) of VPS9D1-AS1 was synthesized by Genechem, Shanghai, China. All the shRNA sequences are as follows: shVPS9D1-AS1-1: TGGCGTCAGCTCTCTGGAAAT; shVPS9D1-AS1-2: CGGCTCTACCACTGTTACTTA; and shVPS9D1-AS1-3: CACCAGAGGAGTCTCTCTCAT. Seventy-two hours after infection, the cells were screened using puromycin at a concentration of 1.6 μg/mL for Eca109 and at 2 μg/mL for TE-13.

### Cell Counting kit-8 (CCK-8) assay

The transfected cells were seeded into 96-well plates (3×10^3^/well), followed by the addition of CCK-8 reagent (Beyotime, China) according to the manufacturer's instructions every 24 h for 5 days. The cell proliferation rate determined by measuring the optical density values at 450 nm using a microplate reader (Molecular Devices, BioTek, USA).

### Cell migration and invasion assays

To evaluate the migration and invasion abilities of ESCC cells, the Transwell chamber (Corning, USA) was used. Matrigel (Invitrogen, USA) was diluted with RPMI-1640 at a 1:9 ratio. After being stably transfected, the cells were resuspended to a density of 10^6^/ml. Then, 100 μl of the resuspension was added into the upper chamber. After incubating for 24 h, the cells remaining on the upper membrane were removed, fixed, stained, and counted under an inverted fluorescence microscope (EVOS FL Auto; Invitrogen).

### Wound healing assay

The confluent transfected cells were scratched with a 1 ml pipette tip, followed by the replacement of serum-free medium. Wound healing was observed and recorded under a fluorescence microscope (EVOS FL Auto; Invitrogen). The Image J software was used to analyze the migration ability.

### Flow cytometric analysis

The prepared transfected cells were stored in 70% ethanol at -20 °C. After washing the cells with phosphate buffered saline, propidium iodide was added to the cells, followed by incubation for 40 min. Then, cell cycle analysis was performed using the BD FACSCalibur system (BD, USA).

### Western blotting

Details of the specific experimental procedure conducted were as previously described in a similar paper 8. GAPDH was used as the control. Anti-CDK4 (ab199728), anti-CDK6 (ab124821), anti-cyclin D1 (ab134175), anti-β-catenin (ab134175), and anti-c-Myc (ab185656) were purchased from Abcam (Cambridge, UK). The agonist of Wnt/β-catenin signaling pathway CT99021 was purchased from Selleck (Selleck Chemicals, USA).

### Xenograft assays in nude mice

Four-week-old female BALB/C nude mice were purchased from the Animal Center of Nantong University and were used in the next study. TE-13 cells, which were stably infected with the lentivirus or negative control, were subcutaneously inoculated into experimental group and control group of nude mice. After 3 weeks the mice were sacrificed and the tumor tissues were stripped and weighed.

### Immunohistochemistry (IHC) analysis

The ENVISION method was used for IHC analysis. Anti-Ki-67 (ab92742) was purchased from Abcam. First, the specimens from the transplanted tumors were fixed, embedded, and cut into 5 μm thick slices. Then, the slices were deparaffinized, hydrated, blocked with 3% H_2_O_2_, and sequentially incubated with specific primary antibodies and horseradish peroxidase-conjugated secondary antibodies. Finally, five low power fields and high power fields were randomly selected for scoring the staining results by two independent observers. The number of positive cancer cells expression <5% was negative (-); 5%~25% was weakly positive (+); 25%~50% was moderately positive (++) and ≥50% was strongly positive (+++).

### Colony formation assay

Approximately 15 ml of the cell suspension containing 2,000 transfected cells was planted into 10 cm petri dishes. After 12 days of incubation, the fixed cells were stained with methyl violet. The number of colonies (≥50 cells) was counted with naked eyes.

### Statistical analysis

The SPSS 23.0 and GraphPad Prism 7.0 software were used to conduct all statistical analyses. All data are expressed as mean ± standard deviation. Between-group variances were calculated using the t-test, chi-square test, and nonparametric test (Wilcoxon test). Overall survival (OS) and progression-free survival (PFS) were calculated by the Kaplan-Meier method using the log-rank test. A *p*-value of less than 0.05 was considered significant.

## Results

### Analysis of lncRNA expression via RNA-sequencing

**Figure 1:**
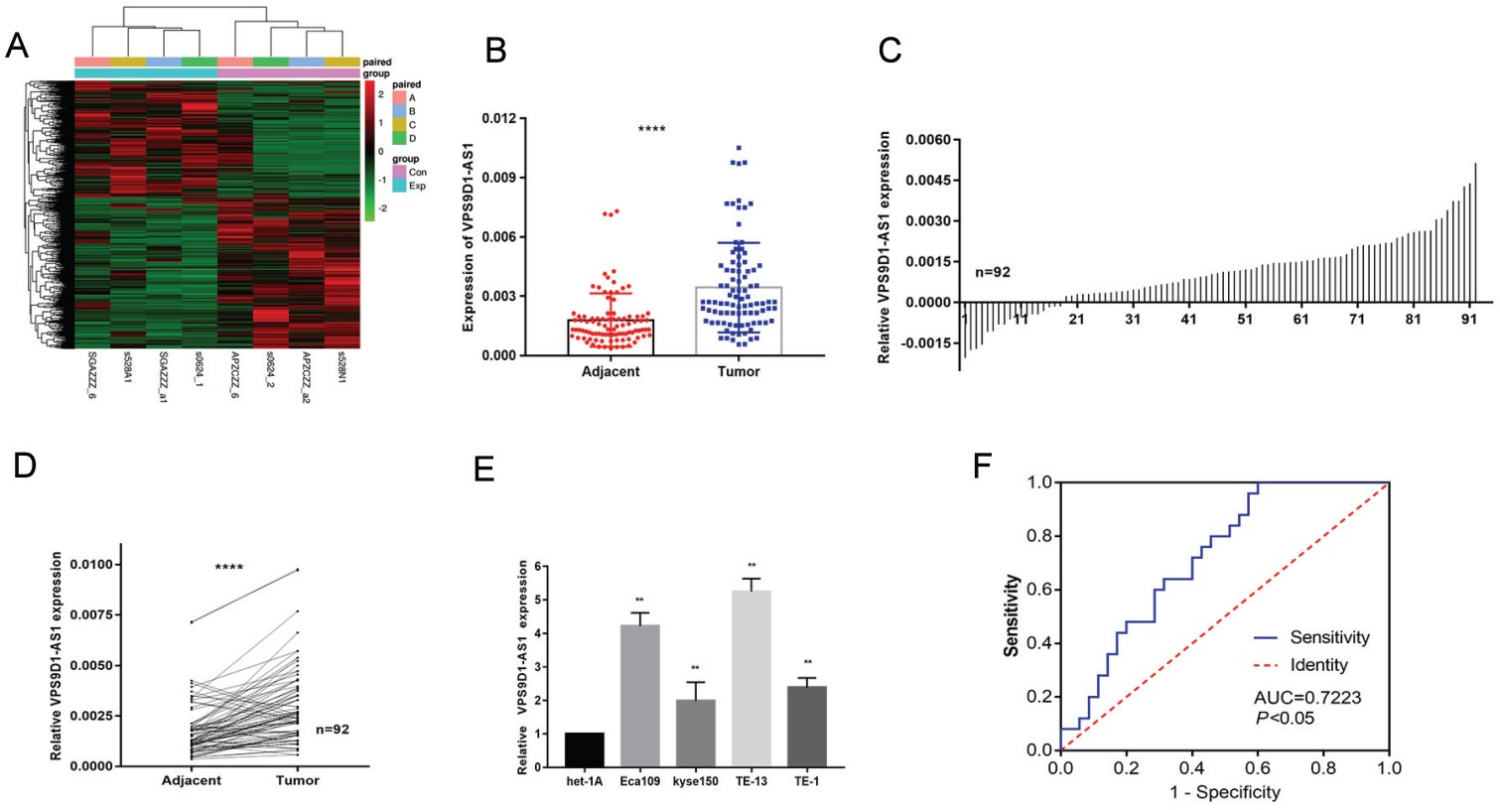
** VPS9D1-AS1 was upregulated in ESCC. (A)** Differential expression profile of lncRNAs. Red represents the high expression value, while green represents the low. **(B-D)** VPS9D1-AS1 was significantly higher in ESCC tissues samples when compared with their matched NATs by qRT-PCR. **(E)** The expression of VPS9D1-AS1 in ESCC cell lines Eca109, Kyse150, TE-13 and TE-1 were increased compared with that in normal esophageal epithelial cells. **(F)** (C) ROC curve of patients based on VPS9D1-AS1 expression in ESCC plasma samples and plasma samples with esophageal benign lesions (*P<.05, **P<.01, ***P<.001, and ****P<.0001).

Based on the sequencing results (*p* < 0.05 and log2 fold change >2 or < 0.5), 3,514 lncRNAs were differentially expressed in ESCC tumors and matched NATs, among which 2,079 were upregulated and 1,435 were downregulated. All different lncRNAs were ranked based on their log2 fold change values. First, we removed lncRNAs with longer than 4000 nucleotides and then eliminated the molecules that have already been studied in esophageal cancer such as H19, SNHG6, LUCAT1, HOXA11. Through the above screening, we obtained several candidate molecules, which were verified using TCGA and ENCORI databases. Finally, VPS9D1-AS1 was selected among these lncRNAs for further studies (**Fig. 1A**). It can be detected in nucleus, cytoplasm, exosomes, and plasma, according to the RNAlocate database.

### Upregulation of VPS9D1-AS1 expression in ESCC tissues, cells and plasma

The expression levels of VPS9D1-AS1 were detected in 92 paired ESCC tissue samples and NATs by conducting a series of qRT-PCR assays. The assays revealed that VPS9D1-AS1 was markedly upregulated in tumor tissues compared with NATs (**Fig. 1B-D**). We further detected VPS9D1-AS1 expression in ESCC cell lines, covering Eca109, Kyse150, TE-1, TE-13, and the esophageal epithelial cell line het-1A. The expression level of VPS9D1-AS1 was higher in all ESCC cell lines than that in het-1A (**Fig. 1E**). Among the cell lines, TE-13 and Eca109 with higher VPS9D1-AS1 expression levels were selected for further studies. Besides that, the expression of VPS9D1-AS1 in 60 plasma samples including 25 patients with ESCC and 35 patients with esophageal benign lesions was detected; then, ROC curve analysis was conducted. The area under the ROC curve was 0.7223 (95% CI: 0.5958-0.8488; *p*=0.0035) (**Fig. 1F**). As a good early diagnostic indicator, the detection of VPS9D1-AS1 in plasma is a noninvasive and convenient method. However, the expression of VPS9D1-AS1 in ESCC plasma is lower than that in ESCC tissues; therefore, further analysis is warranted to determine whether it is a potential diagnostic biomarker.

### Relevant clinicopathologic factors of VPS9D1-AS1 in ESCC

The expression levels of VPS9D1-AS1 in tumor tissues were classified into low-expression and high-expression groups according to the median values. Furthermore, age, gender, smoking history, tumor location, histopathological grade, invasion depth, lymph node metastasis, and tumor-node-metastasis (TNM) stages were recorded, and their correlation with VPS9D1-AS1 expression was determined (**Tables 1 and 2**). VPS9D1-AS1 expression level was an independent factor affects PFS and OS of patients with ESCC.

### High expression of VPS9D1-AS1 leads to poor prognosis

**Figure 2:**
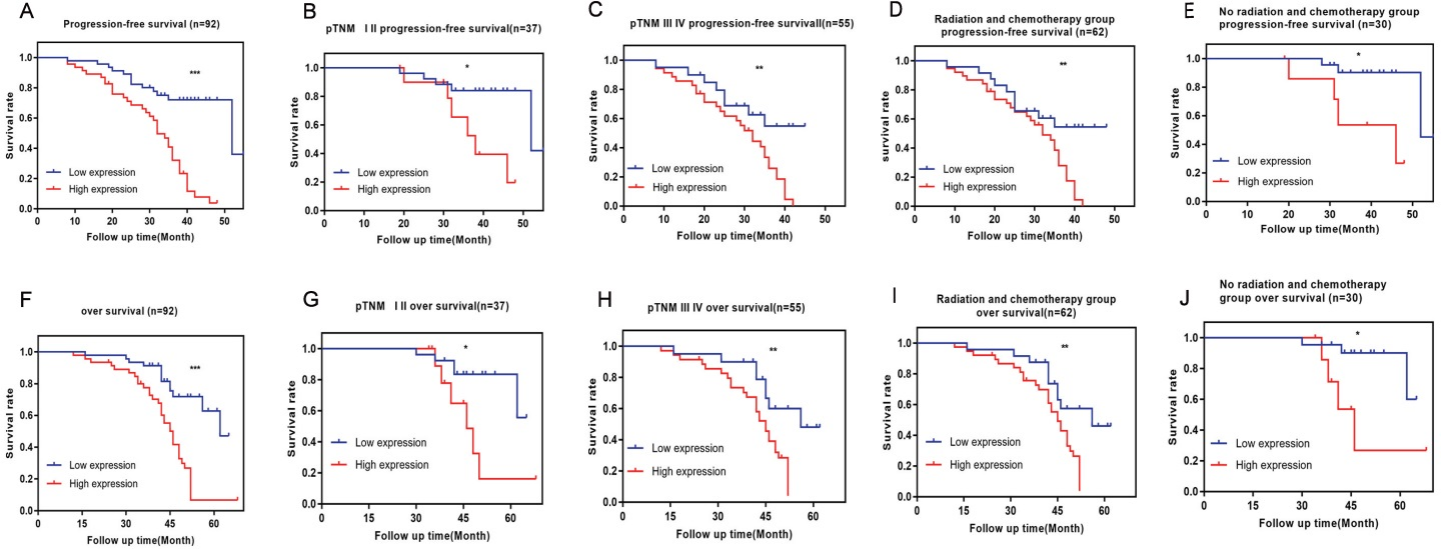
** The prognostic significance of VPS9D1-AS1 in ESCC patients.** Kaplan-Meier analysis of progression-free survival (PFS) based on VPS9D1-AS1 expression in all 92 patients (**A**). Kaplan-Meier analysis of PFS based on VPS9D1-AS1 expression in ESCC patients in stages I-II (**B**), III-IV (**C**) and radiotherapy or chemotherapy (**D**), no radiotherapy or chemotherapy (**E**). Kaplan-Meier analysis of overall survival (OS) based on VPS9D1-AS1 expression in all 92 patients (**F**). Kaplan-Meier analysis of OS based on VPS9D1-AS1 expression in ESCC patients in stages I-II (**G**), III-IV (**H**) and radiotherapy or chemotherapy (**I**), no radiotherapy or chemotherapy (**J**). (*P<.05, **P<.01, ***P<.001, and ****P<.0001).

The results showed that the patients in the high-VPS9D1-AS1 expression group had a shorter PFS and OS than those in the lower-expression group (**Fig. 2A,F**). Further, VPS9D1-AS1 expression was strongly correlated with PFS and OS in advanced clinical stages (stages III and IV) (**Fig. 2B,G**). In patients with early clinical stage, no significant difference was observed in PFS and OS between the high and low expression groups (**Fig. 2C,H**). The short- term PFS and OS of patients without radiotherapy or chemotherapy after undergoing surgery were not significantly different; however, the long-term PFS and OS of those with high VPS9D1-AS1 expression significantly decreased (**Fig. 2E,J**).

Tumor invasion depth and lymph node metastasis were the factors affecting PFS and OS. These clinical variables were considered as potential predictors of survival in univariate and multivariate analyses (**Tables 3 and 4**).

### Knockdown of VPS9D1-AS1 negatively affects the proliferation, migration, and invasion of ESCC cells

**Figure 3:**
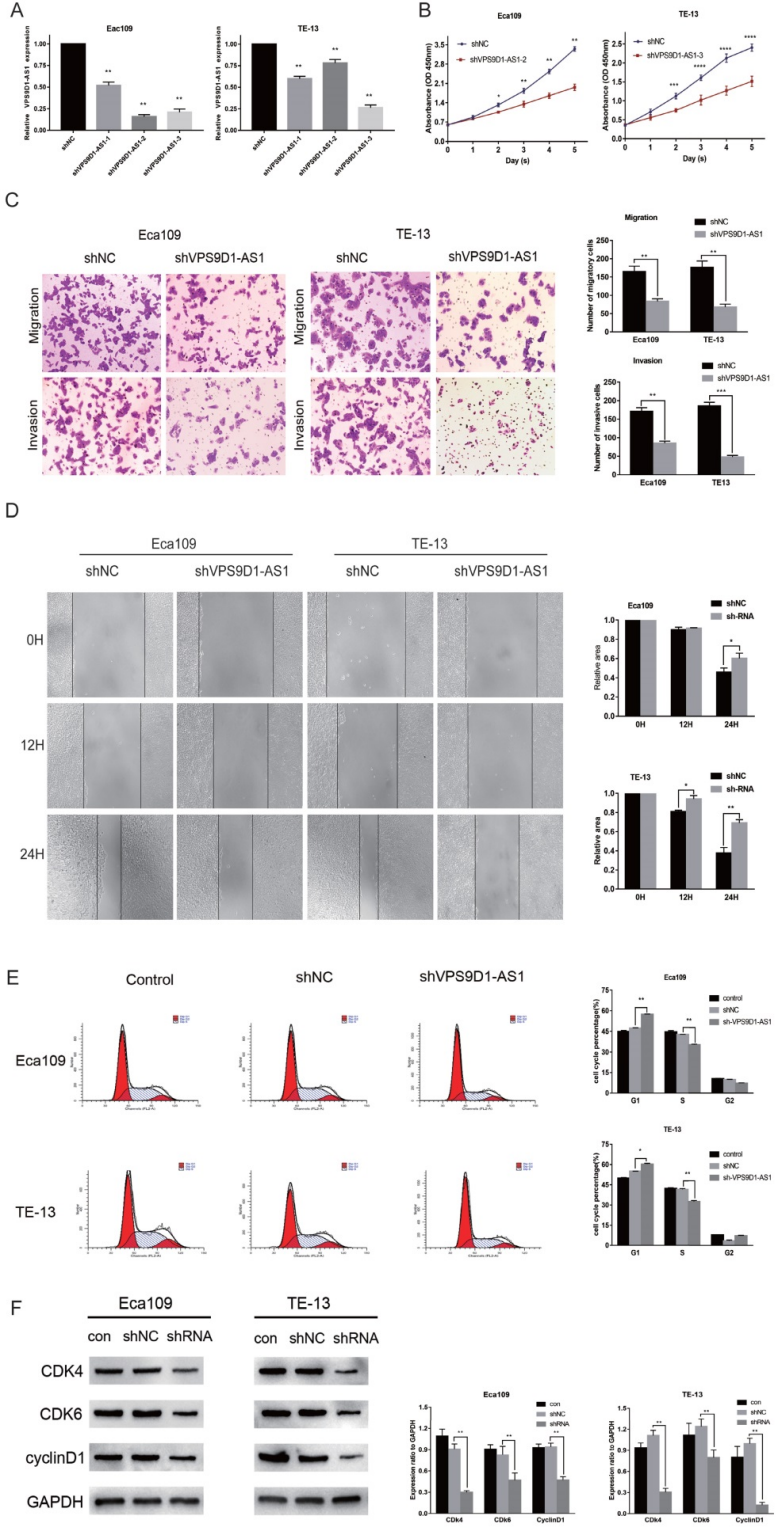
The downregulation of VPS9D1-AS1 expression regulates cell proliferation, invation, migration, mobility and cell cycle. **(A)** Interference efficiency of sh-VPS9D1-AS1-1,2,3 in ESCC cells was evaluated by qRT-PCR.** (B)** CCK-8 assays were performed that VPS9D1-AS1 knockdown significantly inhibited the proliferation of Eca109 and TE-13 cells. **(C)** Transwell assays exhibited that VPS9D1-AS1 knockdown significantly inhibited the invation, and migration of Eca109 and TE-13 cells. **(D)** Wound healing assays were performed that VPS9D1-AS1 knockdown significantly inhibited the mobility of Eca109 and TE-13 cells. **(E)** VPS9D1-AS1 knockdown resulted in cell cycle arrest in Eca109 and TE-13 cells. **(F)** VPS9D1-AS1 knockdown resulted in decreased expression of cell cycle critical proteins in Eca109 and TE-13 cells. (Magnification: 200×,*P<.05, **P<.01, ***P<.001, and ****P<.0001).

Three shRNAs targeting the human VPS9D1-AS1 (shVPS9D1-AS1-1, shVPS9D1-AS1-2, and shVPS9D1-AS1-3) and a nonspecific scrambled shRNA (shNC) were synthesized. Subsequently, they were transfected into Eca109 and TE-13 cells. The unprocessed cells served as a blank control. All shVPS9D1-AS1s significantly suppressed the expression of endogenous VPS9D1-AS1. The efficiency of the three shRNA candidates was measured via qRT-PCR. shVPS9D1-AS1-2, which decreased the level of VPS9D1-AS1 expression by 85%, was better than the other RNAs in Eca109 cells. Meanwhile, shVPS9D1-AS1-3 further decreased the expression level of VPS9D1-AS1 in TE-13 cells (**Fig. 3A**). Thus, these two shRNAs were used in the subsequent analysis.

In the CCK-8 assay, the proliferation of Eca109 and TE-13 cells were significantly impaired after VPS9D1-AS1 knockdown compared with the negative control (**Fig. 3B**).

In the Transwell assay, the number of cells penetrating through the filter was remarkably decreased following VPS9D1-AS1 knockdown in Eca109. Similar results were observed in TE-13 cells. Consistent with the abovementioned findings, the invasiveness of these two cell lines following VPS9D1-AS1 knockdown was also respectively repressed (**Fig. 3C**). Furthermore, VPS9D1-AS1 depletion caused a delay in wound healing in Eca109 and TE-13 cells (**Fig. 3D**). Taken together, the results demonstrate that VPS9D1-AS1 downregulation remarkably restrains the proliferation, migration, and invasion of ESCC cells.

### VPS9D1-AS1 regulates the cell cycle

Cell cycle progression following VPS9D1-AS1 downregulation was significantly impeded at the G0/G1 phase in Eca109 and TE-13 cell lines (**Fig. 3E**). Next, we examined cell cycle regulators, such as CDk4, CDk6, and cyclin D1. All of them were markedly suppressed by VPS9D1-AS1 silencing (**Fig. 3F**). Therefore, we speculate that VPS9D1-AS1 downregulation suppresses tumor growth and progression by inducing cell cycle stagnation.

### Downregulation of VPS9D1-AS1 inhibits ESCC tumorigenesis *in vivo*

**Figure 4:**
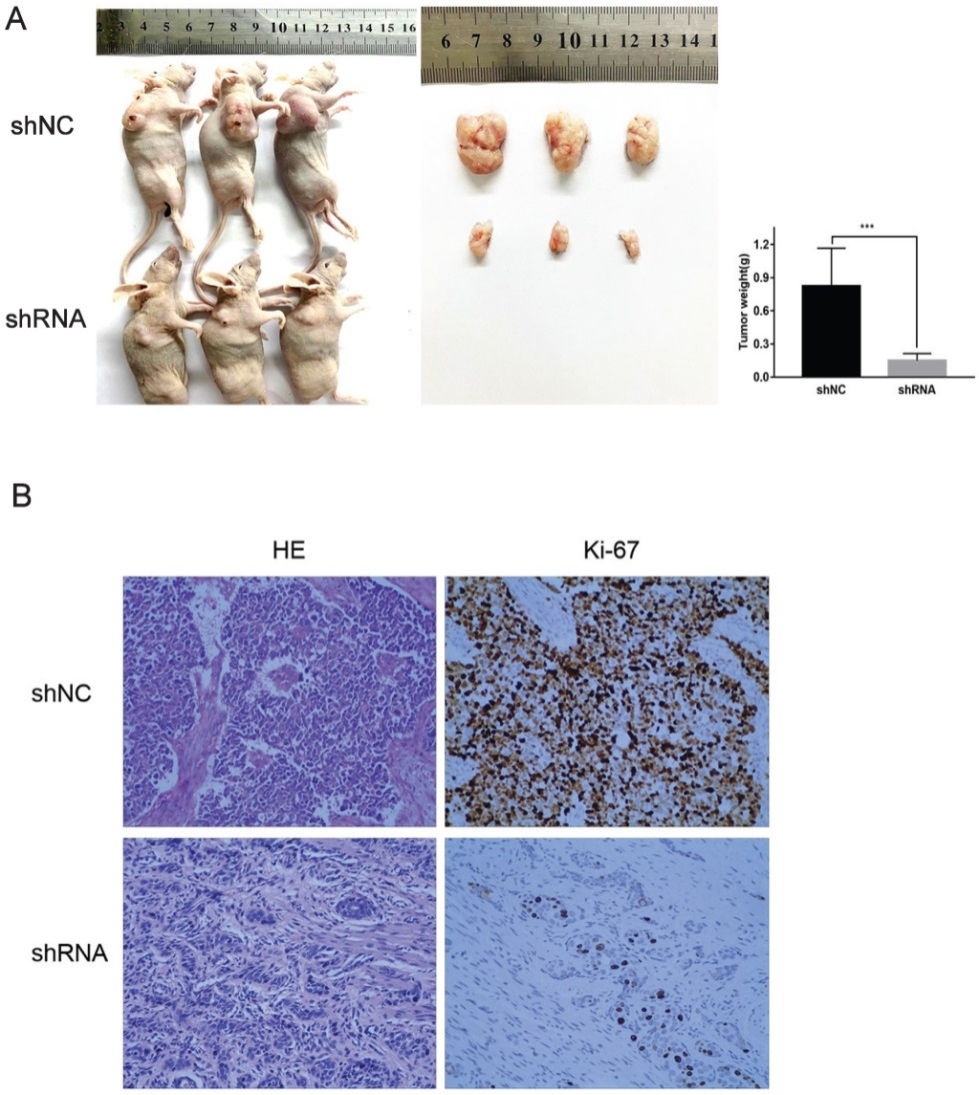
** VPS9D1-AS1 promoted ESCC cell progression *in vivo*. (A)** Knockdown of VPS9D1-AS1 inhibited the proliferation ESCC cells *in vivo*. **(B)** Immunohistochemical staining demonstrated that suppression of VPS9D1-AS1 inhibited the aggressive phenotype of ESCC cells *in vivo*, as indicated by the expression of Ki67-positive cells. (*P<.05, **P<.01, ***P<.001, and ****P<.0001).

With the downregulation of VPS9D1-AS1, tumors that were stripped from the nude mice were remarkably smaller. Further, the index of proliferation, Ki-67, was significantly downregulated, as revealed by IHC analysis. In conclusion, the results suggest that VPS9D1-AS1 promotes tumor growth (**Fig. 4A,B**).

### VPS9D1-AS1 affects ESCC via the Wnt/β-catenin signaling pathway

**Figure 5:**
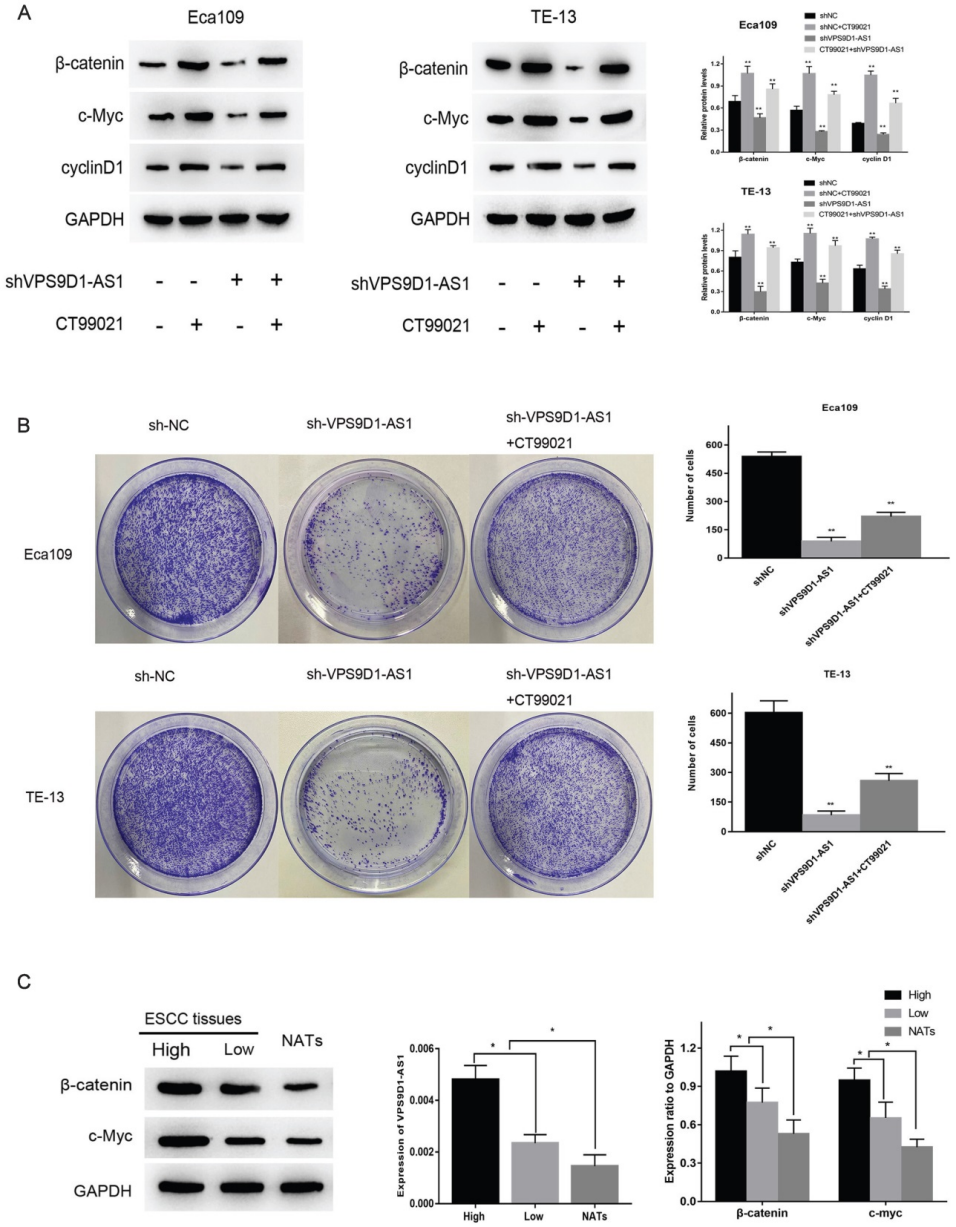
** VPS9D1-AS1 affects ESCC via the Wnt/β-catenin signaling pathway. (A)** Knockdown of VPS9D1-AS1 reduced the expression of key proteins in Eca109 and TE-13 cells, while Wnt/β-catenin pathway agonist CT99021 alleviated this effect. **(B)** Knockdown of VPS9D1-AS1 inhibited the colony‐forming ability in Eca109 and TE-13 cells, while CT99021 alleviated this effect. **(C)** The expression of VPS9D1-AS1 was positively correlated with β-catenin and c-Myc in ESCC tissue samples. (Data are mean±SD.*P<.05, **P<.01, ***P<.001, and ****P<.0001).

Through the enrichment analysis of VPS9D1-AS1, we obtained the most likely related biological function of this molecule, the top five were ribosome biogenesis, RNA modification, translation, ribosome assembly and cell cycle. Among several common pathways associated with tumor invasion and migration, Wnt and c-myc, key molecules in the Wnt/β-catenin pathway, were involved in regulating ribosome biogenesis. Therefore, we speculated that VPS9D1-AS1 may affect the occurrence and development of ESCC via this pathway. To further verify this hypothesis, we detected the expressions of key proteins in this pathway by Western blot. The expressions of these proteins decreased significantly after the downregulation of VPS9D1-AS1, but increased subsequently after the addition of Wnt/β-catenin pathway agonist CT99021 (**Fig. 5A**). The results suggested that VPS9D1-AS1 may affect the proliferation of ESCC cells by regulating the Wnt/β-catenin signaling pathway. In the colony formation assay, VPS9D1-AS1 knockdown in Eca109 and TE-13 cells greatly suppressed colony growth compared with that in control cells, however, when CT99021 was added into shVPS9D1-AS1 group, it was found that the ability of clone formation and growth was significantly improved (**Fig. 5B**).

Moreover, we found that the expression of VPS9D1-AS1 positively correlated with that of β-catenin and c-Myc in ESCC tissue samples. Western blotting revealed that the expressions of β-catenin and c-Myc were significantly higher in ESCC tissues than in NATs. Furthermore, in the high-VPS9D1-AS1 expression group, β-catenin and c-Myc expressions were higher in ESCC tissues compared with those in the low VPS9D1-AS1 expression group (**Fig. 5C**). Taken together, these results demonstrate that VPS9D1-AS1 might promote ESCC progression by regulating the Wnt/β‐catenin signaling pathway.

## Discussion

Previous studies have confirmed the number of lncRNAs associated with the cancer prognosis 9-11. Those studies have indicated that lncRNAs have high potential as a biomarker for cancer diagnosis or prognosis. LncRNAs, miRNAs, and target genes form an interactive regulatory network, in which lncRNAs function as “sponges” or “ceRNAs” 12-14. Although a part of the mechanism of ESCC occurrence and development has been discovered, researchers have found no specific and sensitive biomarkers for early diagnosis and treatment, which have a central impact on improving the survival rate of ESCC.

Several studies have shown that lncRNAs play a carcinogenic or inhibitory role in the occurrence and development of ESCC 4-7. They are usually differentially expressed in ESCC and play a central role in the regulation of epigenetics, as well as in the transcriptional regulation and post-translational modification of proteins 15. LncRNAs have been detected in a variety of microenvironments. They are found not only in tissues but also in serum, plasma, body fluid, and the cerebrospinal fluid 16. LncRNAs such as HOTAIR or XIST may function as a scaffold to recruit proteins involved in chromatin remodeling or histone modification 17-18. LncRNA CCAT2 and ESCCAL-1 have been found to be highly expressed in ESCC tissues; their expression is closely related to the degree of lymph node metastasis and TNM stage. The high expression of these lncRNAs increases the risk of death and shortens survival time in patients 19-20.

VPS9D1-AS1, an antisense RNA that is 1,637 nucleotides long, was initially identified as a lncRNA regulated by c-Myc in colon cancer, as reported by Yoshihiro et al. Therefore, it was named lncRNA MYU (c-Myc-upregulated lncRNA) 21. In patients with gastric cancer, the expression of VPS9D1-AS1 negatively correlates with tumor size and TNM stage 22. The present study further indicated that patients with gastric tumor with low VPS9D1-AS1 expression are expected to show a shorter OS and disease-free survival than those with high VPS9D1-AS1 expression 22. In contrast, VPS9D1-AS1 is overexpressed in prostate and colorectal cancers 23-24. These conflicting findings prompted our interest in accelerating research on VPS9D1-AS1 in ESCC.

The present study demonstrated that VPS9D1-AS1 was upregulated in ESCC tissues, plasma, and ESCC cell lines; the expression level of VPS9D1-AS1 was directly correlated with clinicopathologic features. The present study further showed that VPS9D1-AS1 downregulation inhibited the proliferation, colony formation, migration, invasion, and cell cycle of ESCC cells. In addition, VPS9D1-AS1 expression was positively correlated with β-catenin and c-Myc in ESCC samples.

A previous study has suggested that VPS9D1-AS1 is the direct lncRNA target of the Wnt/c-Myc pathway and participates in the tumorigenicity of colon cancer cells 21. Furthermore, Kawasaki has demonstrated that VPS9D1-AS1 associates with the RNA-binding protein heterogeneous nuclear ribonucleoprotein-K to stabilize CDK6 expression, thereby promoting the G1/S transition of the cell cycle. Is there a similar effect in ESCC? Previous studies have shown that tumor invasion and migration in ESCC are closely regulated by activating the Wnt/β-catenin pathway 25-26. Through the enrichment analysis of VPS9D1-AS1, we obtained the most likely related biological function of VPS9D1-AS1; the top five biological functions were ribosome biogenesis, RNA modification, translation, ribosome assembly, and cell cycle. Wnt and c-Myc co-regulate ribosome biogenesis 27. Previous studies have reported that Wnt signaling regulates c-Myc via Wnt/STOP and the Wnt/β-catenin pathway. The subsequent research thus established the key role of Wnt signaling in ribosome biogenesis via two routes. One is via c-Myc, which is regulated both via Wnt-driven c-Myc expression and via the Wnt/STOP pathway. The other route is c-Myc independent and is a downstream effect of Wnt signaling on the transcription of ribosomal genes 21.

In the present study, a significant decrease in the levels of c-Myc and cyclin D1 proteins were observed following VPS9D1-AS1 silencing in Eca109 and TE-13. This result indicates that VPS9D1-AS1 and c-Myc might form a feedback loop to regulate each other; by inference, we found that VPS9D1-AS1 displays carcinogenicity by regulating the expression of c-Myc in ESCC. It is well-known that Wnt/β-catenin signaling induces the expression of the transcription factor c-Myc, leading to cell proliferation and tumorigenesis. c-Myc is one of the most commonly activated oncogenes and is reported to be involved in many human cancers. Thus, much effort has been devoted to explore the potential mechanisms with the role of c-Myc in the cell cycle. It has been reported that c-Myc induced cell proliferation is generally associate with an increase in CDK4 and CDK6 activities, which regulated G1 progression in colon cancer cells 21. Furthermore, it has been reported that c-Myc induces the expression of CDK4 by directly binding to the CDK4 promoter region. Moreover, c-Myc also regulates the mRNA expression of CDK6. β-catenin is widely distributed in different cells as a multifunctional protein and plays a vital role in cell proliferation, migration, apoptosis, and tumorigenesis 28-30. β-catenin expression was found to be visibly declined when VPS9D1-AS1 was downregulated in Eca109 and TE-13 cells. β-catenin is considered a key point in the regulation of intracellular signal transduction via the Wnt signaling pathway. In conclusion, the present study showed that with VPS9D1-AS1 silencing, the expression of CDK4 and 6 and cyclin D1 declined sharply, but increased subsequently after the addition of Wnt/β-catenin pathway agonist CT99021. However, our experiment has two drawbacks. First, the detection of VPS9D1-AS1 expression in clinical samples was not plenitude. Hence, more samples should be used in future studies to further validate our findings. Second, we speculate that VPS9D1-AS1 promotes tumor progression by regulating the cell cycle via Wnt/β-catenin signaling pathway. However, the specific relative gene and ceRNA network analyses have not been completed. Therefore, its specific mechanism still needs to be explored in the future.

## Conclusions

The present study showed that the level of VPS9D1-AS1 plays a significant role in tumor progression. First, we proved that VPS9D1-AS1 is overexpressed in ESCC tissues, plasma, and cell lines, and that higher expression is often associated with worse prognosis. Furthermore, VPS9D1-AS1 promotes the proliferation and invasion of ESCC cells and also influences the cell cycle via the Wnt/β‐catenin signaling pathway. Overall, this study demonstrates that VPS9D1-AS1 might be a good potential marker for the early diagnosis and prognosis of ESCC.

## Figures and Tables

**Table 1:** Correlation between VPS9D1-AS1 expression and the clinicopathological characteristics of ESCC

Clinical parameter	VPS9D1-AS1		Chi-square test
	Low No. case (n=46)	High No. case (n=46)	*p*-value
**Age (years)**			
<60	21	25	0.404
≥60	25	21	
**Gender**			
Male	37	36	0.797
Female	9	10	
**Smoking history**			
No	26	21	0.297
Yes	20	25	
**Location**			
Upper	2	2	0.977
Middle	26	25	
Lower	18	19	
**Histopathological grade**			
G1	10	3	0.008 *
G2	29	24	
G3	7	19	
**Tumor invasion depth**			
T1	4	1	0.045*
T2	23	14	
T3	14	19	
T4	5	12	
**Lymph node metastasis**			
N0	26	11	0.005 *
N1	11	11	
N2	7	19	
N3	2	5	
**TNM stage**			
I	9	2	0.008 *
II	17	9	
III	15	22	
IV	5	13	

**Table 2:** Spearman-correlation between VPS9D1-AS1 expression and the clinicopathological characteristics of ESCC

Parameter	VPS9D1-AS1 expression level	
	Spearman correlation	p value
Age	-0.052	0.623
Gender	-0.011	0.916
Smoking history	-0.027	0.799
Tumor location	0.11	0.295
Histopathological grade	0.332	0.001*
Tumor invasion depth	0.362	<0.001*
Lymph node Metastasis	0.443	<0.001*
TNM stage	0.435	<0.001*

**Table 3:** Univariate and multivariate Cox regression analyses of VPS9D1-AS1 for progression-free survival (PFS) of patients in the study cohort (n = 92)

Variable	PFS		
	HR	95%CI	*p* value
**Univariate analysis**			
Age	0.958	0.536-1.711	0.884
Gender	1.193	0.592-2.406	0.622
Smoking history	1.238	0.694-2.208	0.47
Tumor location	0.988	0.592-1.647	0.962
Histopathological grade	3.291	1.991-5.441	<0.001*
Tumor invasion depth	3.369	2.222-5.110	<0.001*
Lymph node metastasis	2.977	2.107-4.204	<0.001*
TNM stage	3.669	2.406-5.687	<0.001*
Expression of VPS9D1-AS1	4.727	2.379-9.390	<0.001*
**Multivariate analysis**			
Histopathological grade	0.858	0.399-1.847	0.695
Tumor invasion depth	1.136	0.478-2.698	0.773
Lymph node metastasis	1.524	0.754-3.083	0.241
TNM stage	1.931	0.670-5.572	0.223
Expression of VPS9D1-AS1	2.461	1.161-5.281	0.019*

**Table 4:** Univariate and multivariate Cox regression analyses of VPS9D1-AS1 for overall survival (OS) of patients in the study cohort (n = 92)

Variables	OS		
	HR	95%CI	*p* value
**Univariate analysis**			
Age	1.198	0.667-2.149	0.545
Gender	1.176	0.583-2.371	0.651
Smoking history	1.293	0.725-2.306	0.385
Tumor location	0.976	0.605-1.575	0.921
Histopathological grade	2.268	1.396-2.384	0.001*
Tumor invasion depth	2.075	1.403-3.069	<0.001*
Lymph node metastasis	1.918	1.405-2.618	<0.001*
TNM stage	2.128	1.455-3.113	<0.001*
Expression of VPS9D1-AS1	4.195	2.110-8.341	<0.001*
**Multivariate analysis**			
Histopathological grade	0.914	0.423-1.977	0.820
Tumor invasion depth	1.167	0.495-2.749	0.724
Lymph node metastasis	1.338	0.694-2.579	0.384
TNM stage	1.206	0.410-3.544	0.733
Expression of VPS9D1-AS1	3.071	1.476-6.391	0.003*
